# Comparing Machine Learning and PLSDA Algorithms for Durian Pulp Classification Using Inline NIR Spectra

**DOI:** 10.3390/s23115327

**Published:** 2023-06-04

**Authors:** Dharma Raj Pokhrel, Panmanas Sirisomboon, Lampan Khurnpoon, Jetsada Posom, Wanphut Saechua

**Affiliations:** 1 Department of Agricultural Engineering, School of Engineering, King Mongkut’s Institute of Technology Ladkrabang, Bangkok 10520, Thailand; 64601186@kmitl.ac.th (D.R.P.); panmanas.si@kmitl.ac.th (P.S.); 2 School of Agricultural Technology, King Mongkut’s Institute of Technology Ladkrabang, Bangkok 10520, Thailand; lampan.kh@kmitl.ac.th; 3 Department of Agricultural Engineering, Faculty of Engineering, Khon Kaen University, Khon Kaen 40002, Thailand; jetspo@kku.ac.th

**Keywords:** Monthong durian, multivariate classification algorithms, dry matter content, soluble solid content, NIR spectroscopy, Partial Least Squares Discriminant Analysis (PLS-DA), machine learning, neural network

## Abstract

The aim of this study was to evaluate and compare the performance of multivariate classification algorithms, specifically Partial Least Squares Discriminant Analysis (PLS-DA) and machine learning algorithms, in the classification of Monthong durian pulp based on its dry matter content (DMC) and soluble solid content (SSC), using the inline acquisition of near-infrared (NIR) spectra. A total of 415 durian pulp samples were collected and analyzed. Raw spectra were preprocessed using five different combinations of spectral preprocessing techniques: Moving Average with Standard Normal Variate (MA+SNV), Savitzky–Golay Smoothing with Standard Normal Variate (SG+SNV), Mean Normalization (SG+MN), Baseline Correction (SG+BC), and Multiplicative Scatter Correction (SG+MSC). The results revealed that the SG+SNV preprocessing technique produced the best performance with both the PLS-DA and machine learning algorithms. The optimized wide neural network algorithm of machine learning achieved the highest overall classification accuracy of 85.3%, outperforming the PLS-DA model, with overall classification accuracy of 81.4%. Additionally, evaluation metrics such as recall, precision, specificity, F1-score, AUC ROC, and kappa were calculated and compared between the two models. The findings of this study demonstrate the potential of machine learning algorithms to provide similar or better performance compared to PLS-DA in classifying Monthong durian pulp based on DMC and SSC using NIR spectroscopy, and they can be applied in the quality control and management of durian pulp production and storage.

## 1. Introduction

Durian (*Durio zibethinus*) is a tropical fruit grown in Southeast Asia and is highly appreciated by consumers throughout Asia [1]. Thailand is the leading exporter of durian fruit in terms of volume and exports them to different countries [2]. The exports of Thai durian have been consistently growing each year [3]. There are three main varieties of durian, namely Monthong, Chanee, and Kanyao. Among these three varieties of durian, Monthong is the most well-known exported variety of durian [4].

Dry matter content (DMC) and soluble solid content (SSC) are two major parameters used in the evaluation of the quality of durian pulp. The DMC is an indication of the fruit’s maturity [5], and the SSC is an indication of its ripeness [6]. Durian fruit at different stages of maturity have different sensory qualities. When the fruit become mature, they start to ripen. When the immature durian is ripe, it has a reduced flavor and taste, while, if it is overripe, it will decay rapidly after harvest. When fruit growth starts to slow down, the formation of sugars, starch, lipids, DMC, and carbohydrates takes place. The DMC of durian is mainly due to the accumulation of starch, while the SSC is created by reducing and non-reducing sugars [7]. The maturity and the DMC and SSC of durian pulp show a linear relationship; when the maturity increases, the DMC and SSC increase [8].

The measurement of these parameters is very important for commercial purposes. The price of durian varies according to these two parameters. In addition, the classification of durian on the basis of these two parameters is essential for commercial purposes because it allows them to be classified into different groups on the basis of these parameters, as they represent the quality of the fruit. The traditional method of measuring these two parameters is destructive, so it cannot be used for commercial purposes. According to the Thai Agricultural Standard (TAS) 3-2013, the harvesting time of Monthong durian fruit is between 105 and 110 days after anthesis (DAA) [9]. Fruits harvested at different DAA have different maturity levels, which is directly related to the quality of the fruit. Traditionally, durian maturity is assessed using a combination of methods, such as counting the DAA, evaluating the sound of the pitch after tapping, checking the stem flexibility, and observing the spike tip color [10,11]. However, these traditional methods of classification have several limitations that make them unsuitable for modern durian production. One major issue is the subjective nature of the classification, which relies on the experience and judgement of individual classifiers, leading to inconsistent classifications. This subjectivity can be influenced by various factors, such as personal biases, environmental conditions, and cultural preferences, which can compromise the accuracy of classification. Furthermore, the traditional methods can be time-consuming and labor-intensive as they require a close examination of each fruit, which is problematic for large-scale durian production, where time and efficiency are critical. This can result in delays and inefficiencies that impact the overall productivity and profitability of durian production.

Recently, research has been conducted on the topic of improving the non-destructive maturity classification model for durian fruit using near-infrared spectroscopy. Authors have investigated the use of near-infrared spectroscopy (NIRS) and machine learning algorithms to improve the non-destructive maturity classification of durian fruit. In a study, two NIRS spectrometers were used to scan durian fruit at different maturity stages, and three supervised machine learning algorithms were tested. The results showed that the use of both rind and stem spectra provided the greatest efficiency, with the LDA model exhibiting the highest accuracy (training accuracy = 97.28%, test accuracy = 96.25%). The authors suggested that NIR spectroscopy has the potential to be used for the non-destructive estimation of durian maturity and could be used for quality control in the durian export industry [12].

Due to its real-time monitoring capability, cost-effectiveness, and time efficiency, the inline measurement of fruit constituents and classification using NIR spectra has gained significance. Extensive research has been conducted on the measurement of fruit properties and classifying fruit based on such properties. A Vis–SWNIR spectrometer was integrated into a conveyor system to measure the inline DMC and SSC of durian pulp [6]. The spectra of the samples were acquired in two orientations, scanning upright pulps from 2018 and stable pulps from 2019, and the optimal model showed $R^{2}$ values of 0.88 for DMC calibration, 0.83 for DMC prediction, 0.70 for SSC calibration, and 0.70 for SSC prediction, with an RMSEP of 4.32% and 4.0%, respectively, and a RPIQ of 3.52 for DMC and 2.2 for SSC prediction [6]. Several other studies have explored the application of the inline measurement of NIR spectra for the analysis of different fruit, such as apple, orange, pear, strawberry, and mango, including the classification and prediction of specific constituents [13,14,15,16,17]. The results obtained from these studies provide clear evidence that the utilization of the inline measurement of NIR spectra is effective in achieving accurate classification and the precise prediction of specific constituents.

In the study titled “Hyperspectral Images-Based Crop Classification Scheme for Agricultural Remote Sensing”, a pixel-based approach utilizing Principal Component Analysis-Based Edge-Preserving Features (PCA-EPF) and an SVM classifier was introduced. This approach successfully improved the crop classification accuracy in hyperspectral remote sensing, achieving results of over 90% in the performance metrics measured across nine different crop types [18].

Previous research presented promising results in determining the quality parameters (DMC and SSC) of durian pulp through inline measurement. This study aimed to classify durian pulp based on DMC and SSC into three classes (mature, moderately mature, and immature) in real time, using an inline spectrometer and a conveyor system for efficient and accurate results. The models were built using five spectral preprocessing techniques: Savitzky–Golay and Standard Normal Variate (SG+SNV), Moving Average and Standard Normal Variate (MA+SNV), Savitzky–Golay and Mean Normalization (SG+MN), Savitzky–Golay and Baseline Correction (SG+BC), and Savitzky–Golay and Multiplicative Scatter Correction (SG+MSC). Both PLSDA and optimizable machine learning algorithms were applied in the Classification Learner app of MATLAB, including neural networks, ensembles, k-nearest neighbors, support vector machines, Naive Bayes, discriminants, and trees. The classification results from each algorithm were compared. This model can accurately classify durian into different maturity stages, ensuring consistent quality and reducing the risk of selling underripe or overripe fruit. Additionally, it helps to increase the production capacity, meet the market demand, and expand durian businesses.

Partial Least Squares (PLS) was originally developed as a latent variable modeling technique, primarily used for linear regression analysis [19]. PLS was later extended to include PLS Discriminant Analysis (PLS-DA) for classification purposes [20], which is capable of analyzing small sample sizes and handling multicollinearity in data, particularly in cases such as chemical spectroscopy data. These types of data are often high-dimensional and exhibit strong correlations among neighboring independent variables. PLS addresses the issue of high-dimensional data by employing latent projections before modeling, thereby mitigating the challenges associated with the curse of dimensionality [21]. PLSDA has limitations with regard to effectively capturing and modeling complex data and nonlinear relationships [22,23]. Machine learning has attracted extensive interest due to its capability to effectively model complex nonlinear data patterns [24,25,26]. Numerous studies have been conducted comparing PLSDA with machine learning algorithms for classification tasks [27,28,29,30]. In the majority of these studies, machine learning algorithms have demonstrated superior performance compared to PLSDA.

The objective of this research is to identify and select machine learning algorithms that outperform others. The method is compared with the PLSDA algorithm in terms of performance parameters such as accuracy, precision, recall, specificity, F1-score, area under the receiver operating curve, kappa, and Matthews correlation coefficient. By evaluating and comparing these performance measures, this study aims to determine the most effective algorithm for the classification of durian pulp. Furthermore, another objective is to explore the feasibility of classifying durian pulp based on DMC and SSC using inline measurement. By investigating this possibility, the study aims to contribute the knowledge of durian pulp classification techniques, particularly in the context of inline measurement. The findings of this research can potentially be utilized to auto-grade durian pulp into three stages of maturity: mature, moderately mature, and immature.

## 2. Materials and Methods

### 2.1. Sampling

The study utilized a total of 415 Monthong durian pulp samples, which were sourced from a factory and subsequently sent to the NIRS Research Center for Agricultural Products and Food within the Department of Agricultural Engineering, School of Engineering, King Mongkut’s Institute of Technology Ladkrabang. Four distinct experiments were conducted on these samples, with data collection taking place on 31 May 2021, 16 August 2021, 31 August 2021, 15 October 2021, and 15–16 June 2022. The samples were collected at 110 DAA and were visually classified by experienced gardeners, based on their levels of maturity. The classification system used in this study consisted of three levels of maturity, designated as A, B, and C, and three stages of ripening, designated as 1, 2, and 3. Specifically, “A” corresponded to full maturity, “B” corresponded to moderate maturity, and “C” corresponded to immature samples. Additionally, “1” corresponded to full ripening, “2” corresponded to moderate ripening, and “3” corresponded to unripe samples. Prior to transportation to the laboratory, the samples were prepared overnight and packaged in food-grade plastic to reduce moisture loss. Spectral data were collected at the NIR Spectroscopy Research Center for Agricultural Products and Food within the Department of Agricultural Engineering at King Mongkut’s Institute of Technology Ladkrabang in Bangkok, Thailand, where the ambient temperature was approximately 25 ± 2 °C in the morning. The samples were also measured for DMC and SSC after scanning.

**Figure 1:**
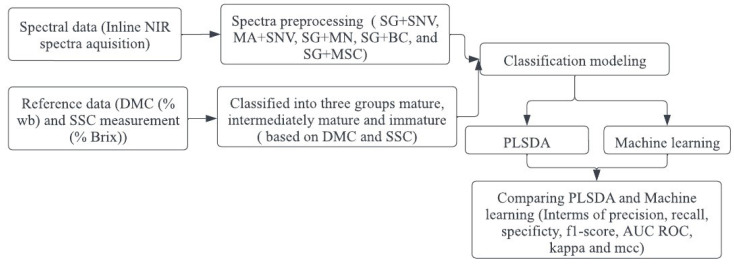
Flow chart diagram for classification modeling.

Figure 1 illustrates the flowchart representing the classification modeling process used in this study.

### 2.2. Inline NIR Scanning

**Figure 2:**
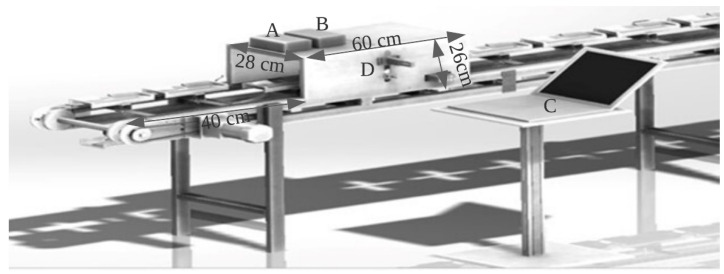
NIR scanning set-up (A = spectrometer, B = light source, C = PC, and D = proximity sensor).

The inline near-infrared (NIR) scanning process involved collecting spectra through inline measurement using an AvaSpec-2048-USB2 standard fiber-optic spectrometer (Avantes, Apeldoorn, The Netherlands) with a wavelength range of 300–1160 nm and a spectral resolution of 2.4 nm. The optical bench utilized a 75 mm focal length and the detector used was a CCD linear array with 2048 pixels. The AD converter had a 16-bit length with a 2 MHz sampling frequency and a sample speed with onboard averaging of 1.1 ms/scan. The light source was an Ava Light-HAL Standard 10 W tungsten halogen lamp, and a compact stabilized halogen fan-cooled light source was used in the visible and NIR range, having a wavelength range of 350–2500 nm. A fiber-optic reflection probe (FCR7IR200-2-BX) consisting of seven fibers with a 200 µm core, six light fibers, and one read fiber in two separate legs enclosed in a silicon inner tube was used, as well as a flexible stainless-steel connector with an SMA connected to the light source and spectrometer to obtain the spectral information of the sample. Each pulp was kept in an upright position in a black plastic tray for stability and placed on a chain conveyor with a speed of 0.17 m/s. The tray speed was adjusted using a speed controller unit with power of 60 watts. To sort the trays according to the grade of the pulp, an oil-free air compressor was used, with a maximum pressure of 0.8 MPa and a fan speed of 1480 r/min. The light source was activated 15–30 min before collecting the white and dark spectra, to allow it to warm up. To correct for the influence of the unstable intensity of the light source on the spectra and to eliminate the background noise within the detector, the Teflon reference material spectrum, or a white reference, was acquired. To obtain the dark and reference scans in one run, the time required for the detector to capture the radiation, also known as the integration time, was set at 4.5 ms, such that the maximum value of reflectance from the reference material (Teflon) was taken over the wavelength range of around 90 of the full analog-to-digital converter (ADC) scale. The optimum focal distance, i.e., the distance between the sensor and the sample surface, was approximately 2.5 cm. However, as the sample size varied, the focal length was altered. As the sample approached the black box, the proximity sensor was placed along the same vertical plane, and when the sample reached the sensor boundary in the black box, consisting of the spectrometer and light source, the sensor sent a signal to the spectrometer to start scanning as shown in Figure 2. Each spectrum was obtained from an average of 200 scans throughout the longitudinal top surface of the durian pulp.

### 2.3. Reference Analysis

#### 2.3.1. Soluble Solid Content (SSC) Measurement

After the completion of inline near-infrared (NIR) spectra collection, the SSC of the samples was determined using a refractometer (PAL-1, S/No L218454, Atago, Tokyo, Japan) with the technical specifications listed in Table 1.

The SSC of each sample was measured in longitudinal sections throughout the scanning area, with the measurements reported in terms of percent Brix. To ensure accurate results, the refractometer was cleaned with distilled water prior to each measurement. Three measurements were taken at the head, middle, and bottom positions of each durian sample, and the refractometer was cleaned with distilled water and dried with tissue paper after each measurement. The average SSC value was then calculated from the measurements taken at the head, middle, and bottom sections of each durian sample.

#### 2.3.2. Dry Matter Content (DMC) Measurement

The dry matter content (DMC) of the pulp samples was determined by homogenizing a portion of the scanned pulp that had undergone soluble solid content (SSC) measurement. The homogenized samples were then placed in aluminum moisture cans with a 5 cm diameter and 3 cm height. Approximately 5 g of the durian sample was taken and placed in the aluminum moisture can. The initial weight of the sample was determined using a high-precision electronic balance (Mettler Toledo Model- JS1203C, Columbus, OH, USA). The samples were then placed in a controlled-environment oven (Memmert GmbH, Model-30-1060, Schwabach, Germany). The temperature of the oven was set at 60 °C for 24 h. The weight of the sample was measured at 3 h intervals until a constant weight was obtained. The DMC of the sample was calculated by subtracting the moisture content, determined by the weight loss during the drying process, from the initial weight of the sample and reported as a percentage of the sample’s dry weight (% wb).

### 2.4. Separation into Classes Using Classification Criteria

The durian pulps were separated into three classes, namely mature, moderately mature, and immature, based on the classification criteria established by the Durian Meat Export Company. These criteria were determined from the DMC and TSS values, as outlined in Table 2. As discussed earlier, the samples were evaluated using an electronic balance (Mettler Toledo Model JS1203C) and refractometer (Atago PAL-1, Japan) to determine their DMC and TSS values, respectively. The classification criteria were applied by comparing the measured DMC and SSC values of the samples against the established standards. The samples that met the established standards for DMC and SSC were classified as mature, moderately mature, or immature accordingly.

### 2.5. Dataset Information

The dataset used in this study consists of near-infrared (NIR) spectra obtained from a spectrometer, along with reference values for dry matter content (DMC) and total soluble solids (TSS), obtained using an electronic balance and refractometer, respectively. The data were transferred to Microsoft Excel for further analysis.

Data Structure in the Excel Spreadsheet: The first row contains the titles “DMC” and “SSC” in the first and second columns, respectively. The third column represents the categorical variable showing the group to which each sample belongs. Starting from the fourth column, each column corresponds to a wavelength ranging from 450 nm to 1000 nm. Each subsequent row, from the second row to the 416th row, represents a sample, where the first column contains the DMC value for the respective sample, the second column contains the TSS value for the respective sample, the third column represents the categorical variable for the respective sample, and the remaining columns contain the absorbance values of the NIR spectra for each sample at their respective wavelengths.

### 2.6. Different Algorithms for Classification

#### 2.6.1. Partial Least Squares Discriminant Analysis

Partial Least Squares Discriminant Analysis (PLS-DA) is a supervised classification algorithm that uses the PLS regression method and linear discriminate method to discriminate a dataset into different classes [31]. PLS establishes the relationship between the predictor variables and response variables using a reduced number of latent variables. Latent variables maximize the covariance between predictor variables and response variables. Then, LDA takes the latent variable as the input to make class predictions. For binary classification, PLS1 is used, where the response variable is either 0 or 1, depending on whether it belongs to the given class or not [32,33]. For the PLS2 method, if there are G number of classes and N number of samples, then we set the response variable as (N × G) with a dummy variable [34,35]. There are several PLS-DA methods used for different purposes. They include Standard PLS-DA, Orthogonal Partial Least Squares Discriminant Analysis (OPLS), Sequential Inner–Outer Model PLS Discriminant Analysis (SIMPLS-DA), Robust Partial Least Square Discriminant Analysis (Robust PLS-DA), and Multivariate Curve Resolution–Partial Least Squares Discriminant Analysis (MCR-PLS-DA). The mathematical steps needed to perform PLSDA can be summarized as follows. First, the partial least squares regression method is used to identify the set of latent variables (LVs) that explain the maximum amount of variation in both *X* and *Y*. The mathematical expression for PLS regression can be represented as

(1) $$X=TP^{T}+E$$

(2) $$Y=UQ^{T}+F$$

where *T* is a matrix of *X* scores, *P* is a matrix of *X* loadings, *U* is a matrix of *Y* scores, *Q* is a matrix of *Y* loadings, *E* is a matrix of *X* residuals, and *F* is a matrix of *Y* residuals. The *LV*s are extracted from the PLS regression model by multiplying *X* by the normalized loading matrix $P_{normalized}$.

(3) $$LV=X*P_{n}ormalized$$

The extracted *LV*s are then used as input to a linear discriminant analysis (LDA) model, which separates the samples into different classes based on their class membership. The mathematical expression for LDA can be represented as

(4) $$Y^{\prime }=W^{\prime }*LV+B$$

where $Y^{\prime }$ is the predicted class membership, $W^{\prime }$ is the weight vector and is obtained by constrained optimization, and *B* is the bias term.

#### 2.6.2. Artificial Neural Network (ANN)

Artificial neural networks are a specific type of machine learning algorithm that are inspired by the structure and function of the human brain [36,37,38,39]. The human nervous system can learn from the past, and, in a similar way, ANNs are able to learn from the data and provide responses in the form of predictions or classifications. An ANN consists of an input layer, output layer, and hidden layer. The purpose of the input layer is to receive the input data, the hidden layer processes the input data, and the output layer computes the final prediction. The number of neurons in the input nodes is equal to the number of explanatory variables in the input data. Each neuron in the input layer corresponds to the explanatory variable. It does not perform any computation; it passes the input data to another layer. The value of each neuron is set to the corresponding value of each explanatory variable in the input data. There may be one or more hidden layers located between the input layer and output layer. Each neuron in the hidden layer receives input from the neurons in the previous layer, processes it using a nonlinear activation function, and sends it to the neurons in the next layer. Values entered in hidden node are multiplied by weights, and then weighted inputs are summed to produce a single number [40,41]. Increasing the number of hidden layers and the number of neurons in the hidden layer increases the performance of the model. However, too many hidden layers and neurons in the hidden layer may cause overfitting, where the model shows good performance with training data and poor performance with new data. The different activation functions used include Sigmoid, Rectified Linear Unit (ReLU), hyperbolic tangent (Tanh), Leaky ReLU, Exponential Linear Unit ELU), and Softmax. ReLU is a commonly used activation function [42,43]. The output layer is the final layer of the artificial neural network (ANN) and computes the final prediction. In supervised learning, the output layer has one neuron for each class, while, in regression, it has one neuron. The activation function used for classification in the output layer is Softmax, and, for regression, the linear activation function is used. A wide neural network consists of only one hidden layer with a large number of neurons in a single layer. In deep neural networks, there are multiple hidden layers. The mathematical computation inside hidden layers can be summarized as the output of the activation function in the hidden layer:

(5) $$Z=\sum_{i=1}^{n}\left(W_{i}X_{i}+b\right)$$

The model identified the hyperparameter where the model was optimized. Then, the model was trained on the optimized hyperparameter. The results of the trained model were obtained, and then the model was tested to evaluate its performance on a new dataset. Results from the application were obtained in terms of confusion matrices and the receiver operating curve (shown in Table 3).

### 2.7. Software Used for Classification

The classification modeling was performed using a licensed MATLAB R2021b version from MathWorks [44], obtained through KMITL. The machine learning algorithm employed for this purpose was run using the Classification Learner, a built-in application in MATLAB. Additionally, Partial Least Squares Discriminant Analysis (PLS-DA) was performed using the PLS-Toolbox from Eigenvector Research Incorporated [45].

### 2.8. Classification Modeling

After acquiring spectral data, DMC, and SSC from an experiment, they were then transferred to an Excel file. The samples were then sorted into different classes, with a total of 415 durian samples being analyzed. Outliers were identified using Q residuals reduced and Hotelling’s T-squared reduced plots at a significance level of *p* = 0.950. Samples outside the boundary line (critical value) were considered outliers, with 11 samples being identified as such. The remaining 404 samples were used for modeling, with the data being imbalanced in terms of the distribution of samples among the mature (43.31%), moderately mature (50%), and immature (6.68%) classes. Due to the low number of samples belonging to the immature category, our dataset is imbalanced, which may negatively impact the performance of model developed from these data, as the model may struggle to correctly classify samples in the under-populated immature category as it does not have sufficient data to learn from. The samples were divided into an 80% training set and a 20% test set via the holdout method, with 324 and 80 samples being used in each set, respectively. Classification was performed using the PLSDA algorithm, which was implemented using Version 9.1 of the PLS-Toolbox and Solo in MATLAB. Additionally, machine learning algorithms were employed, with the Classification Learner application being used to build the model. A five-fold cross-validation technique was employed in both PLSDA and machine learning, with the sample being divided into five subsets or folds. In this cross-validation procedure, each subset was utilized as a validation set once, while the remaining four subsets served as the calibration set. This process was repeated five times, with different subsets being used for evaluation each time, and the results were averaged to estimate the model’s performance. The dataset was trained using seven different optimizable machine learning algorithms, namely optimizable neural networks, optimizable ensembles, optimizable k-nearest neighbors, optimizable support vector machines, optimizable Naive Bayes, optimizable discriminants, and optimizable trees. Each optimizable algorithm searched for the optimal hyperparameters within a specified range, and the hyperparameters that resulted in the best performance of the model were selected. Table 4 presents the search ranges of the hyperparameters used by each algorithm.

## 3. Results

### 3.1. Spectral Characteristics and Spectral Pretreatment

**Figure 3:**
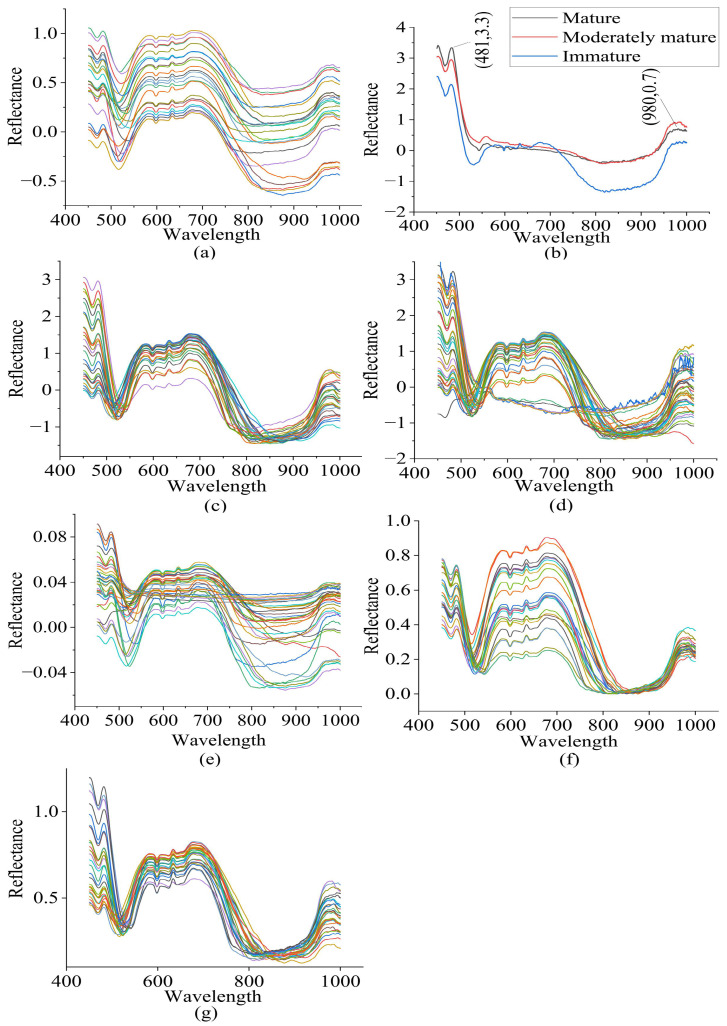
(**a**) Raw spectra, (**b**) average spectra of each group, (**c**) SG+SNV pretreated spectra, (**d**) MA+SNV pretreated spectra, (**e**) SG+MN pretreated spectra, (**f**) SG+BC pretreated spectra, and (**g**) SG+MSC pretreated spectra of durian pulp.

After identifying 11 outlier spectra using a Q residuals reduced (*p* = 0.95) (1.42%) and Hoteling’s T-squared reduced (*p* = 0.950) (98.58%) plot, the remaining 404 spectra were taken for modeling. There was a high level of noise in the raw spectra before 450 nm and after 1000 nm. Hence, spectra in the spectral range between 450 nm and 1000 nm were taken for modeling. The spectral characteristics of the raw spectra in the wavelength range of 450 nm to 1000 nm are visualized in Figure 3. Peaks and valleys can be seen in the raw spectra’s plots. The spectra showed high absorbance in the wavelength range between 450 nm and 481 nm, with a maximum at 481 nm, where bond vibration occurred. Additionally, high absorbance was observed between 550 nm and 715 nm, with another peak noticeable at 980 nm, near the absorbance band of water. The average spectra of each of the three classes were plotted to visualize the spectral characteristics of the three different groups of durian pulp. The raw spectra contained noise and a baseline shift, and there was a large scaling difference between the absorbance bands. In order to remove these defects, the raw spectra were pretreated using different combinations of spectral preprocessing techniques, including MA+SNV, SG+SNV, SG+MN, SG+BC, and SG+MSC.

Two options were available for the smoothing of the spectra: the MA and the SG filters. An MA smoothing method with a window size of 13 data points and an SG smoothing method with a polynomial order of 2, a window size of 13 data points, and a symmetric kernel indicating equal weights on either side were applied. A baseline offset method was applied to correct the baselines of the spectra, and full MSC was applied to remove the scattering effect. The SG filter is a polynomial-based smoothing technique that is more effective than MA in removing noise, while preserving the overall shape of the spectra. In addition, the performance of the model built using SG+SNV was better than that of the model built on MA+SNV in terms of overall classification accuracy, while the performance regarding the other parameters measured was almost similar. Hence, other preprocessing techniques were combined with SG to perform spectral smoothing. In terms of the overall classification rate, the SG+SNV and SG+BC spectral preprocessing methods were found to be the most and second-most effective, respectively.

### 3.2. Outlier Detection

**Figure 4:**
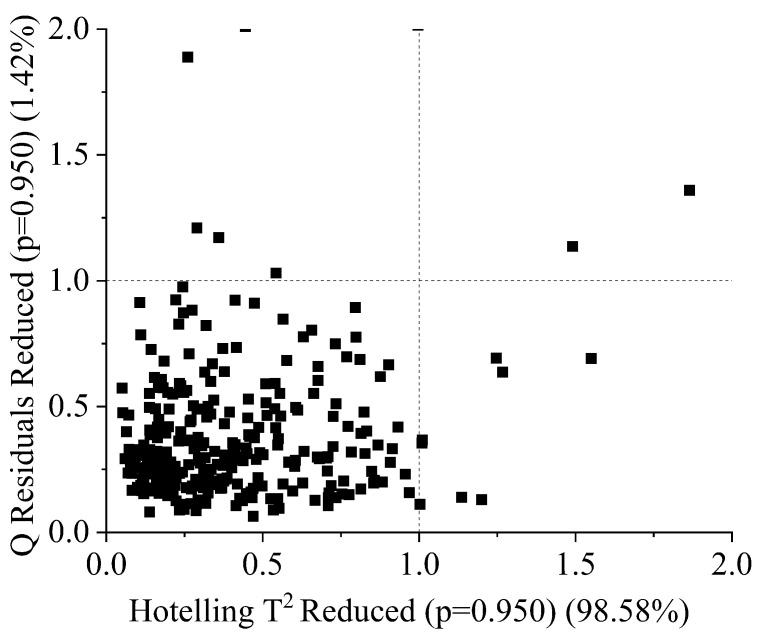
Q residuals (reduced) vs. Hotelling’s T${}^{2}$ (reduced) plot for durian pulp classification using PLSDA and machine learning.

Eleven outliers were detected in the sample using the Q residuals reduced (at a significance level of *p* = 0.95) and Hotelling’s T-squared reduced (at a significance level of *p* = 0.95) statistical methods. The critical values for both Q residuals reduced (at a significance level of *p* = 0.95) and Hotelling’s T-squared reduced (at a significance level of *p* = 0.95) were both 1, which was used as a threshold to identify outliers in the sample. The outliers were found to deviate significantly from the overall pattern of the spectra, as demonstrated by their high Q residuals and Hotelling’s T-squared values, which exceeded the calculated critical values. It could be seen that samples outside the boundary line ( in Figure 4) were outliers; these samples were removed before modeling. Additionally, the removal of these outliers from the sample was found to improve the overall performance of the classification model, as it reduced the potential for bias and increased the overall robustness of the model. This highlighted the importance of identifying and removing outliers prior to building a model, in order to improve the accuracy and reliability of the resulting model.

### 3.3. Classification Modeling

#### 3.3.1. Classification Using PLSDA

A total of 324 samples in the training set and 80 samples in the test set were used to build the model. The modeling process employed five different combinations of spectral preprocessing techniques to obtain the optimum model. The overall accuracy for the model built with the SG+SNV spectral preprocessing technique was 81.43%. In terms of the overall classification accuracy, the model built with the SG+SNV spectral pretreatment technique yielded better results than the models built with the other four spectral preprocessing techniques. The overall accuracy of the models built on 381MA+SNV, SG+MN, SG+BC, and SG+MSC was 78%, 78%, 78%, and 77%, respectively. The total overall accuracy of a classification model can be used as a comparative measure by taking the average of the model’s overall accuracy on the training set (validation) and test set. Thus, the models can be compared based on their predictive performance. The results of these calculations can provide insights into how well the models are able to generalize to new data and can be used to identify potential overfitting or underfitting issues. The comparative analysis of the models based on the test accuracy revealed that the SG+MN model had superior performance compared to the other four models, suggesting its ability to obtain accurate predictions on previously unseen data. Other classification model performance metrics, such as precision, bias, specificity, F1-score, area under the ROC curve (AUC), kappa, and Matthews correlation coefficient (MCC), were calculated and compared for each model. These were good indicators of the performance of the models and provided further insights into the strengths and weaknesses of each model.

The model built using the SG+BC spectral preprocessing technique demonstrated higher performance in terms of recall (82%), precision (83%), and F1-score (83%), indicating its ability to reject samples from other groups while accurately classifying samples of a specific group. The models demonstrated a good balance between recall and precision, suggesting a high level of reliability. All of the models had specificity of 50%, indicating that they performed no better than a random classifier in correctly identifying negative classes. The overall AUC of the ROC curve built on spectra treated with SG+MSC showed a value of 0.85, the highest among the compared models, indicating strong performance in accurately classifying samples at various threshold values. Overall, the AUCs for all other models were in the range of 0.83–0.84, which suggested good performance in accurately classifying positive and negative samples. The model employing SG+SNV spectral preprocessing achieved the highest kappa scores, with values of 0.69 for the training set and 0.60 for the test set. These scores indicated a high level of agreement between the predicted and actual classes for both the training and test datasets, demonstrating the robustness and reliability of this model’s performance. The Matthews correlation coefficient (MCC) was computed by averaging the individual MCC scores between each pair of classes. The range of obtained MCC values was between 0.59 and 0.70, with the model that employed the SG+SNV spectral preprocessing technique yielding the highest MCC score, indicating a good correlation between the classes. The performance metrics of each model are presented in Table 5.

**Figure 5:**
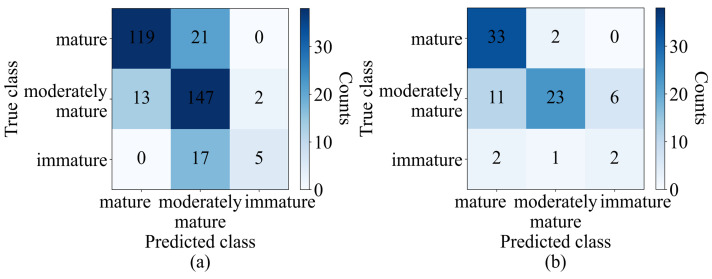
Confusion matrix of (**a**) training set (validation) and (**b**) test set of PLSDA (SG+SNV).

The results of the optimum model obtained from PLSDA were represented in a confusion matrix table, providing a visual representation of the model’s performance for each class. The confusion matrix analyzed the accuracy of the classification model by comparing the predicted class with the actual class. In the training set, 119 out of 140 samples were correctly classified as mature, 147 out of 162 samples were correctly classified as moderately mature, and 5 out of 22 samples were correctly classified as immature. Moreover, 21 samples in the mature group were incorrectly classified as moderately mature, while 13 and 2 of the remaining 15 moderately mature samples were wrongly classified as mature and immature, respectively. Seventeen of the immature samples were wrongly classified as moderately mature. The test set results can also be seen in the confusion matrix table. The accuracy in the training set for the mature, moderately mature, and immature classes was 85%, 90.7%, and 22.7%, respectively. The classification accuracy for the test set was computed as 94.2% for the mature class, 57.5% for the moderately mature class, and 40% for the immature class can be seen in Figure 5.

**Figure 6:**
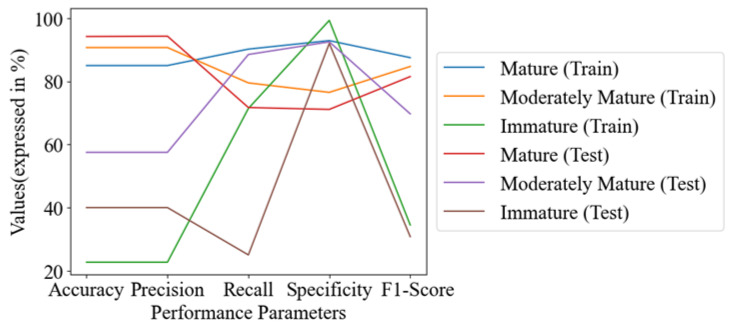
Line chart of performance metrics of PLSDA (SG+SNV).

The performance outcomes of specific groups in the training and test sets were visualized for comparison. In the graphs, each line represented a group in either the training or test set. The mature group had the highest accuracy, with values of 85% in the training set and 94.2% in the test set. The moderately mature group had accuracy of 90.7% in the training set and 57.5% in the test set. The immature group had relatively low accuracy, with values of 22.7% in the training set and 40% in the test set. The precision, recall, and F1-score for both the training and test sets of the mature, moderately mature, and immature groups were in the range of 22.7–94.3%, 25–92.9%, and 30.8–87.5%, respectively. The minimum value was found in the immature group, and the maximum value was found in the mature group, which was nearly equal to that of the moderately mature group. The specificity was found to be in the range of 71.1% to 99.3% for all groups in both the training and test sets (as outlined in Figure 6).

**Figure 7:**
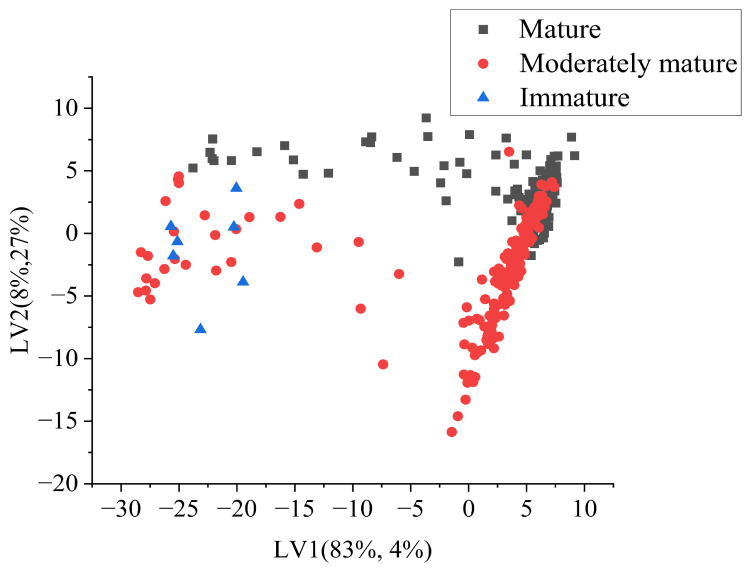
Latent variable score plot from PLSDA (SG+SNV).

Class separation can be observed from the latent variable score plot. The plot shows the distribution of samples in the space defined by the LVs, with the first LV on the x-axis and the second LV on the y-axis. The samples were represented by points, with the colors of the points indicating the class of the sample. Red points represented the moderately mature category, black points represented the mature category, and blue points represented the immature category. From the plot, it was evident that the scores of LV1 played a significant role in separating the classes. Immature durian pulp had low values of LV1, as observed on the left side of the scatter plot, while moderately mature and mature samples showed some overlap on the right side of the score plot (Figure 7).

**Figure 8:**
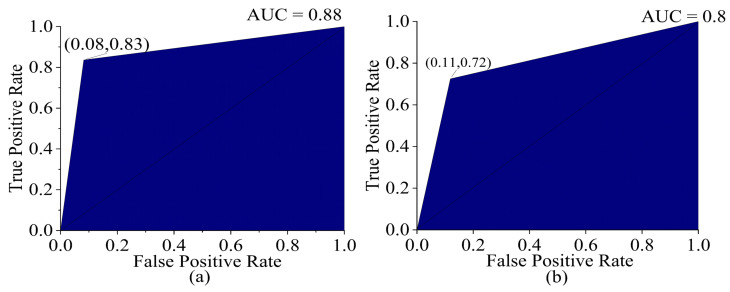
ROC curve (**a**) training set (validation) and (**b**) test set of PLSDA (SG+SNV).

When a dataset contains imbalanced data, it is better to evaluate the AUC ROC curve. To obtain an ROC curve for a three-class classification problem, the micro-average method is applied, as it is suitable for the conversion of the data into a binary classification problem. The optimum operating point on the ROC curve balances the true positive rate (TPR) and the false positive rate (FPR) in both the training set and test set, with a high TPR (0.72–0.83) and low FPR (0.08–0.11), representing a good trade-off in correctly identifying a specific class while rejecting the other classes. The ROC curve was shifted towards the top-left corner of the graph, indicating higher discriminatory power compared to a random classifier, which is represented by the reference line in the ROC plot, which has an AUC value of 0.5. The reference line serves as a benchmark in evaluating the discriminatory power of a classification model, with points above the line indicating a stronger discriminatory ability than random chance and points below this indicating worse performance. The AUCs in the training and test sets were 0.88 and 0.80 (shown in Figure 8), respectively, indicating that the model was better at distinguishing between the positive and negative classes in the dataset.

#### 3.3.2. Classification Using Machine Learning

Among the models trained with the seven optimizable machine learning algorithms, as discussed before, the optimizable ANN gave a higher result in terms of overall accuracy in all five cases of spectral pretreatment. This is due to its ability to capture complex nonlinear relationships, extract relevant features automatically, exhibit robustness to noise, and handle complex decision boundaries. While performing the hyperparameter tuning, our model was optimized with the wide neural network (a type of ANN) in all cases. As in PLSDA, the model built with SG+SNV gave higher overall accuracy of 85.38% (86.1% training and 80% test) with (L = 2, g = ReLU, Z = yes, λ = 0.0018, $n_{1}$ = 154, $n_{2}$ = 3). The effectiveness of SG+SNV in mitigating noise, minimizing baseline shift, reducing scattering effects, and achieving the optimal normalization of the NIR spectra significantly improved the quality and informativeness of the spectra, resulting in enhanced classification accuracy compared to other techniques. Other models’ overall accuracy ranged between 80.2% and 84.6% (training 80.2% to 85.8%, and test 77.5% to 81.2%), which was higher than the result of the PLSDA. In addition, the overall precision, recall, and F1-score values for all models were equal to each other and were in the range of (80% to 85%), with the highest value obtained with SG+SNV and the lowest with SG+BC; in the case of PLSDA, the highest value was obtained with SG+BC (precision = 81%, recall = 82%, F1-score = 82%) and the lowest value with SG+MN (precision = 78%, recall = 79%, and F1-score = 78%). In terms of specificity, for which the value ranged between 68% and 77%, it was higher than that for PLSDA (50% with all models), indicating the stronger ability of the models to reject the samples of other classes. Overall, the AUC ROC, Cohen’s kappa, and MCC were found to be within the range of 0.87–0.92 for training (0.68–0.74), test (0.60–0.67), training (0.79–0.85), and test (0.67–0.72), respectively as presented in Table 6.

Table 7 summarizes the neural network model’s architecture and parameter information, providing essential information for each layer. Each layer is represented by key information, such as the number of neurons present in the layer, the activation function, the regularization value, the weight size, and the bias size. The activation function used in each layer plays a crucial role in determining the behavior of the network. Additionally, the regularization value is included to showcase the level of regularization applied to prevent overfitting. Furthermore, the dimensions of the weight matrices and bias vectors are displayed, providing insights into the size and structure of the network’s parameters. This information helps us to understand the complexity and connectivity of the neural network model.

**Figure 9:**
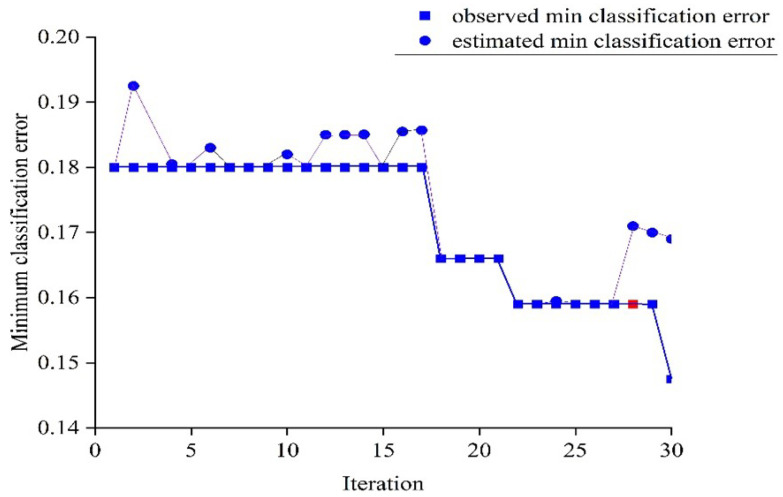
Minimum classification error plot for training of ANN classifier with SG+SNV preprocessing.

The optimization of the optimum model (obtained with the SG+SNV preprocessing technique) was performed through a hyperparameter tuning process that utilized the Bayesian optimizer (with an acquisition function of expected improvement per second and 30 iterations) to search for the best hyperparameters. The ANN model searched for hyperparameters within a defined range, including L = 1–3; activation functions (g) such as ReLU, Tanh, Sigmoid, and none; Z = yes/no; λ = $3.0864\times 10^{-8}-308.642$; and hidden layers ($n_{1}$, $n_{2}$) with 1–300 neurons. The optimal hyperparameters were determined at the 28th iteration and are indicated by the red points in Figure 9, with values L = 2, g = ReLU, Z = Yes, λ = 0.0018, $n_{1}$ = 154, and $n_{2}$ = 3. These hyperparameters were chosen based on the minimization of the upper confidence interval of the classification error objective model, ensuring a low classification error and avoiding overfitting. The observed and estimated minimum classification errors are also displayed in the plot.

**Figure 10:**
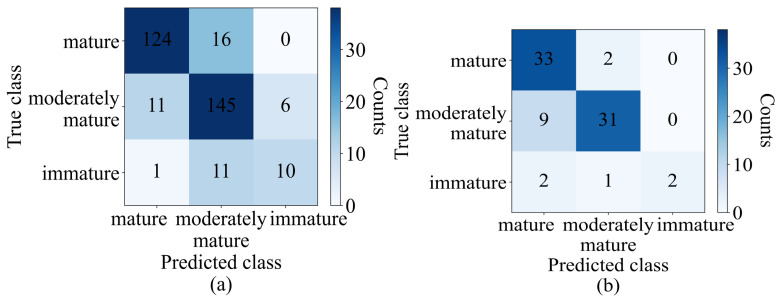
Confusion matrix, (**a**) training set (validation), and (**b**) test set of optimized ANN model.

The model was also compared with PLSDA in terms of the class-wise classification results, as indicated by Figure 10. In the training set, among 140 mature durians, 124 (88.5%) were correctly classified as mature, while 145 samples out of 162 (89.5%) and 10 out of 22 (45.45%) were correctly classified as moderately mature and immature, respectively. In the test set, the number of samples correctly classified as mature, moderately mature, and immature was 94.2%, 77.5%, and 40%, respectively. In comparison with PLSDA, in the training set, five more samples were correctly classified as mature, two fewer samples were correctly classified as moderately mature, and five more samples were correctly classified as immature. In the test set, both the PLSDA and optimum machine learning models both identified 33 (94.2%) and 2 (40%) samples as mature and immature, while PLSDA identified 23 samples as moderately mature, and the machine learning model (optimum model) identified 31 as such.

**Figure 11:**
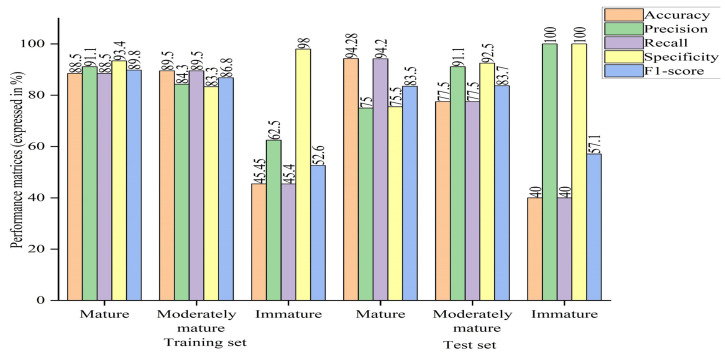
Bar chart of performance metrics of optimized ANN model.

In addition, the model was compared in terms of group-wise performance metrics. A general overview of the bar chart shows higher performance metrics in both the mature and moderately mature groups than the immature group, compared to the case of PLSDA. In terms of the overall accuracy, some groups performed well with PLSDA and some with machine learning (optimized model), as discussed earlier regarding the confusion matrix. In the case of the optimized machine learning model, precision and recall had different values, ranging within 62.5–100% and 22.7–94.3%, where the maximum value was found for the mature group and the minimum value was found for the immature group; the moderately mature group had almost similar results, unlike the case of PLSDA, where the same values were obtained for both. Although the F1-score was balanced between recall and precision, its value was higher for machine learning (optimized model), ranging between 57.1 and 89.8%, where the minimum score was found for the immature group and the maximum score was found for the mature group, while the moderately mature group had a similar value to the mature group, indicating that the model had the ability to correctly classify mature and moderately mature samples, rejecting other classes. In terms of specificity, the machine learning algorithm (optimized model) had a higher or equal value compared to PLSDA, with the exception of the immature group in the training set can be seen in Figure 11.

**Figure 12:**
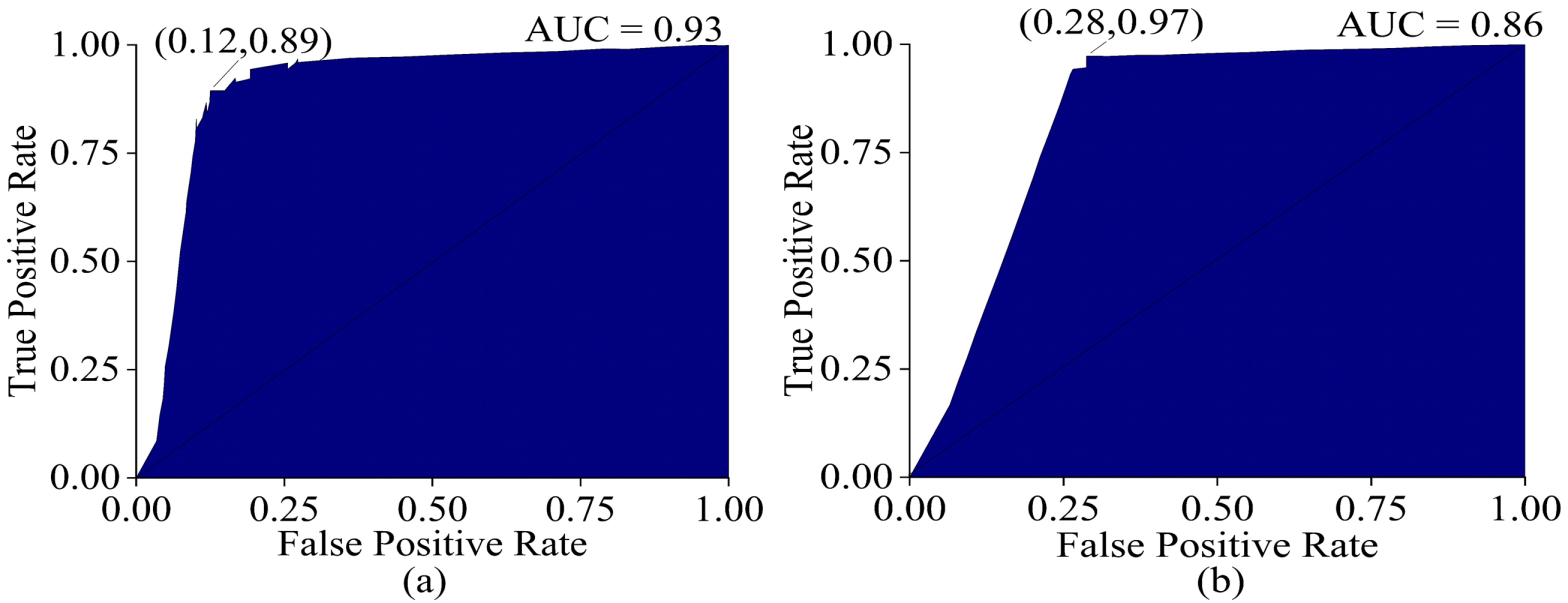
ROC curve (**a**), training set (validation), and (**b**) test set of optimized ANN model.

The ROC curve obtained from machine learning (optimized model) had a higher AUC (training = 0.93, test = 0.86) than PLSDA. The micro-average method was applied to obtain the ROC curves of the three groups, as in the case of PLSDA. The machine learning classifier had a higher TPR (training = 0.89, test = 0.97) compared to PLSDA, which was an advantage. However, it also had a higher FPR (training = 0.12, test = 0.28) compared to PLSDA, which was a disadvantage. This means that while the machine learning algorithm was better at correctly identifying positive classes, it also had a higher rate of false positive identifications, increasing the number of false positive predictions as observed in Figure 12. In comparison, PLSDA had a lower FPR but also a lower TPR.

## 4. Conclusions

In conclusion, this study demonstrated that the machine learning ANN algorithm outperformed the PLSDA algorithm, as well the other machine learning algorithms, with an accuracy rate of 85.3%. The ANN (machine learning) algorithm performed better than the PLSDA algorithm in terms of other performance measures, including recall, precision, specificity, F1-score, AUC ROC, kappa, and MCC. This indicates its suitability for the classification of durian pulp based on DMC and SSC. This highlights the potential of machine learning, particularly ANN, in achieving a good and accurate model for durian pulp classification, while using inline-measured spectra. It also indicates the potential for the automated grading of durian pulp into three categories: mature, moderately mature, and immature. This research provides valuable insights into the use of machine learning and PLSDA algorithms for durian pulp classification using the inline measurement of NIR spectra, serving as a guide for future studies. It highlights the potential of machine learning algorithms in classifying durian pulp for quality control and sorting purposes. Further research should explore larger datasets and different types of durian pulp, along with investigating feature selection techniques to enhance the machine learning algorithm’s performance. Additionally, the use of a balanced dataset could potentially improve the model’s effectiveness.

## Figures and Tables

**Table 1:** Technical specifications of the refractometer (PAL-1, S/No L218454, Atago, Japan).

Measurement Range (% Brix)	Accuracy (% Brix)	Resolution (% Brix)	Measurement Temperature Range (°C)	Dimension (cm)	Weight (g)
0–85	±0.2	0.1	10–100	5.5 × 3.1 × 10.9	100

**Table 2:** Criteria for durian pulp classification based on DMC and SSC.

Class	Number of Samples	Dry Matter Content (%)	Total Soluble Solids (% Brix)
Mature	175	DM ≥ 32	TSS ≥ 28 Brix
Moderately mature	202	DM ≥ 32	22 ≤ TSS < 28 Brix
Immature	27	DM < 32	TSS < 22 Brix

Source: Personal communication with the Durian Meat Export Company.

**Table 3:** Classification performance parameters.

Performance Parameter	Formula
Accuracy	$\frac{\mathrm{TP}+\mathrm{TN}}{\mathrm{TP}+\mathrm{TN}+\mathrm{FP}+\mathrm{FN}}$
Precision	$\frac{\mathrm{TP}}{\mathrm{TP}+\mathrm{FP}}$
Recall	$\frac{\mathrm{TP}}{\mathrm{TP}+\mathrm{FN}}$
Specificity	$\frac{\mathrm{TN}}{\mathrm{TN}+\mathrm{FP}}$
F1-Score	$\frac{2\cdot \mathrm{Precision}\cdot \mathrm{Recall}}{\mathrm{Precision}+\mathrm{Recall}}$
Kappa	$\frac{\mathrm{Po}-\mathrm{Pe}}{1-\mathrm{Pe}}$
Matthews Correlation Coefficient (MCC)	$\frac{TP\times TN-FP\times FN}{\sqrt{\left(TP+FP)(TP+FN)(TN+FP)(TN+FN\right)}}$
Area Under ROC curve	∫TPRd(FPR)

Abbreviations: TP—True Positive; TN—True Negative; FP—False Positive; FN—False Negative; MCC—Matthews Correlation Coefficient; Po—Observed Proportion of Agreement; Pe—Expected Proportion of Agreement.

**Table 4:** Hyperparameter search range for different algorithms using Bayesian optimization.

Algorithms	Hyperparameter Search Range
Optimizable neural network	L = 1–3, g = ReLU, Tanh, Sigmoid, None, Z = yes/no, $\lambda =3.0864\times 10^{-8}-308.642$,
	$n_{1}=1$–300, $n_{2}=1$–300
	Optimizer: Bayesian optimization
	Acquisition function: Expected improvement per second plus
	Iterations: 30
Optimizable SVM	kernel = [’linear’, ’poly’, ’rbf’], degree = [2, 5] (for polynomial function only)
	gamma = [$10^{-5}$, $10^{5}$] (rbf only)
	C1 = [0.01, 100] (for box constraint)
	C2 = [$10^{-5}$, $10^{5}$] (for regularization parameter)
Optimizable ensemble	min_samples_leaf = [0, 1], max_depth = [1, 30], criterion = [“gini”, “error”]
	n_estimators = [10, 500], ensemble_method = [“AdaBoost”, “RusBoost”, “Bag”]
	min_samples_split = [2, … upper limit (50 or 100)], learning_rate = [0, 1]
Optimizable KNN	n_neighbors = [1, 10], metric = [“euclidean”, “manhattan”, “chebyshev”,
	Euclidean City block, Chebyshev, Minkowski (cubic), Mahalanobis,
	Cosine, Correlation, Spearman, Hamming, Jaccard]
	weights = [“uniform”, “distance”], standardized = [Yes, No]
Optimizable tree	n_estimators = [lower limit (10), … upper limit (1000)],
	ensemble_method = [“bagging”, “boosting”, “stacking”], max_splits = [lower limit (1),
	… upper limit (30)], split_criterion = [“gini”, “error”],
	surrogate_splits = [True, False]
Optimizable Naive-Bayes	naive_bayes_algorithm = [“Gaussian”, “Multinomial”, “Bernoulli”],
	regularization_parameter = [0, 1], kernel = [“Gaussian”, “Box”, “Epanechikov”, “Triangle”]
Optimizable discriminant	model = [“LDA”, “QDA”], regularization_parameter = [0, 1] (only for LDA)

**Table 5:** PLSDA classification results using different spectral preprocessing techniques.

Pretreatment	Terminology	Training Set (Validation)	Test Set	Overall
SG+SNV	Number of samples	324	80	
	Accuracy (%)	83.6	72.5	81.4
	Recall (%)	84	73	81
	Precision (%)	84	75	82
	Specificity (%)	50	50	50
	F1-score (%)	84	74	82
	AUC ROC	0.88	0.80	0.84
	Cohen’s Kappa	0.69	0.60	
	MCC	0.70	0.61	
MA+SNV	Accuracy (%)	79.9	76.2	78
	Recall (%)	80	76	79
	Precision (%)	80	77	79
	Specificity (%)	50	50	50
	F1-score (%)	80	77	79
	AUC ROC	0.84	0.82	0.83
	Cohen’s Kappa	0.62	0.60	
	MCC	0.62	0.61	
SG+MN	Accuracy (%)	75.6	80.5	78
	Recall (%)	77	81	78
	Precision (%)	78	81	79
	Specificity (%)	50	50	50
	F1-score (%)	78	81	78
	AUC ROC	0.83	0.85	0.84
	Cohen’s Kappa	0.61	0.65	
	MCC	0.59	0.67	
SG+BC	Accuracy (%)	80.2	75	79.2
	Recall (%)	83	79	82
	Precision (%)	84	79	83
	Specificity (%)	50	50	50
	F1-score (%)	83	79	83
	AUC ROC	0.85	0.83	0.84
	Cohen’s Kappa	0.69	0.59	
	MCC	0.69	0.60	
SG+MSC	Accuracy (%)	83.3	78.7	82.4
	Recall (%)	80	75	79
	Precision (%)	81	78	81
	Specificity (%)	50	50	50
	F1-score (%)	81	76	80
	AUC ROC	0.87	0.83	0.85
	Cohen’s Kappa	0.65	0.59	
	MCC	0.63	0.60	

**Table 6:** ANN classification results using different spectral preprocessing techniques.

Pretreatment	Optimum Hyperparameter	Performance	Training (Validation)	Test	Overall
SG+SNV	L = 2	Accuracy (%)	86.1	80	85.38
	g = ReLU	Recall	0.86	0.82	0.85
	Z = Yes	Precision	0.86	0.82	0.85
	λ = 0.0018	Specificity	0.79	0.70	0.77
	$n_{1}$ = 154	F1-score	0.86	0.82	0.85
	$n_{2}$ = 3	AUC ROC	0.96	0.89	0.92
		Kappa	0.74	0.67	
		MCC	0.82	0.69	
MA+SNV	L = 1	Accuracy (%)	81.8	77.5	80.94
	g = none	Recall	0.84	0.81	0.83
	Z = Yes	Precision	0.84	0.81	0.83
	λ = 0.00061	Specificity	0.76	0.68	0.75
	$n_{1}$ = 7	F1-score	0.84	0.81	0.83
		AUC ROC	0.94	0.89	0.91
		Kappa	0.72	0.65	
		MCC	0.79	0.68	
SG+MN	L = 2	Accuracy (%)	80.2	81.2	80.4
	g = none	Recall	0.83	0.78	0.82
	Z = no	Precision	0.83	0.78	0.82
	λ = 0.00000025	Specificity	0.75	0.66	0.73
	$n_{1}$ = 248	F1-score	0.83	0.78	0.82
	$n_{2}$ = 1	AUC ROC	0.89	0.85	0.87
		Kappa	0.69	0.60	
		MCC	0.79	0.67	
SG+BC	L = 1	Accuracy (%)	85.8	80	84.6
	g = sigmoid	Recall	0.85	0.80	0.82
	Z = no	Precision	0.85	0.80	0.82
	$\lambda =4.0622\times 10^{-4}$	Specificity	0.79	0.68	0.73
	$n_{1}$ = 1	F1-score	0.85	0.80	0.82
		AUC ROC	0.97	0.85	0.91
		Kappa	0.73	0.62	
		MCC	0.85	0.69	
SG+MSC	L = 3	Accuracy (%)	83	80	80.4
	g = sigmoid	Recall	0.80	0.77	0.78
	Z = no	Precision	0.83	0.80	0.81
	λ = 0.000187	Specificity	0.83	0.80	0.81
	n${}_{1}$ = 1	F1-score	0.95	0.87	0.91
	n${}_{2}$ = 49	AUC ROC	0.89	0.85	0.87
	n${}_{3}$ = 1	Kappa	0.69	0.60	
		MCC	0.79	0.67	

**Table 7:** Layer-wise parameters of ANN trained on SG+SNV preprocessed spectra.

Layer	Number of Neurons	Activation Function	Regularization Value	Weight Size	Bias Size
Input Layer	971	None	0.0018	None	None
Hidden Layer 1	154	ReLU	0.0018	(154 × 971)	154
Hidden Layer 2	3	ReLU	0.0018	(3 × 154)	3
Output Layer	3	Softmax	0.0018	(3 × 3)	3

## Data Availability

The data used in this study were generated from four distinct experiments performed by the authors, with a total of 415 samples. During the modeling process, 11 samples were removed as outliers. The data include measurements of total soluble solid content, dry matter content, and near-infrared (NIR) spectra for each sample. The data are available upon request from the corresponding author. Any interested researchers may contact the authors for additional information and to request access to the data.
